# Integrated 5-hydroxymethylcytosine and fragmentation signatures as enhanced biomarkers in lung cancer

**DOI:** 10.1186/s13148-022-01233-7

**Published:** 2022-01-24

**Authors:** Xinlei Hu, Kai Luo, Hui Shi, Xiaoqin Yan, Ruichen Huang, Bi Zhao, Jun Zhang, Dan Xie, Wei Zhang

**Affiliations:** 1 National Frontier Center of Disease Molecular Network, State Key Laboratory of Biotherapy, West China Hospital, Sichuan University, No. 17, Section 3, Renmin South Road, Chengdu, 610041 Sichuan People’s Republic of China; 2 Department of Respiratory and Critical Care Medicine, First Affiliated Hospital The Second Military Medical University, Shanghai, 200433 Shanghai People’s Republic of China; 3 Tailai Inc., Shanghai, 200233 People’s Republic of China

**Keywords:** Cell-free 5hmC sequencing, Cell-free DNA fragmentation, Liquid biopsy, Lung cancer, Cancer diagnosis

## Abstract

**Background:**

Lung cancer is one of most common cancers worldwide, with a 5-year survival rate of less than 20%, which is mainly due to late-stage diagnosis. Noninvasive methods using 5-hydroxymethylation of cytosine (5hmC) modifications and fragmentation profiles from 5hmC cell-free DNA (cfDNA) sequencing provide an opportunity for lung cancer detection and management.

**Results:**

A total of 157 lung cancer patients were recruited to generate the largest lung cancer cfDNA 5hmC dataset, which mainly consisted of 62 lung adenocarcinoma (LUAD), 48 lung squamous cell carcinoma (LUSC) and 25 small cell lung cancer (SCLC) patients, with most patients (131, 83.44%) at advanced tumor stages. A 37-feature 5hmC model was constructed and validated to distinguish lung cancer patients from healthy controls, with areas under the curve (AUCs) of 0.8938 and 0.8476 (sensitivity = 87.50% and 72.73%, specificity = 83.87% and 80.60%) in two distinct validation sets. Furthermore, fragment profiles of cfDNA 5hmC datasets were first explored to develop a 48-feature fragmentation model with good performance (AUC = 0.9257 and 0.822, sensitivity = 87.50% and 78.79%, specificity = 80.65% and 76.12%) in the two validation sets. Another diagnostic model integrating 5hmC signals and fragment profiles improved AUC to 0.9432 and 0.8639 (sensitivity = 87.50% and 83.33%, specificity = 90.30% and 77.61%) in the two validation sets, better than models based on either of them alone and performing well in different stages and lung cancer subtypes. Several 5hmC markers were found to be associated with overall survival (OS) and disease-free survival (DFS) based on gene expression data from The Cancer Genome Atlas (TCGA).

**Conclusions:**

Both the 5hmC signal and fragmentation profiles in 5hmC cfDNA data are sensitive and effective in lung cancer detection and could be incorporated into the diagnostic model to achieve good performance, promoting research focused on clinical diagnostic models based on cfDNA 5hmC data.

**Graphical abstract:**

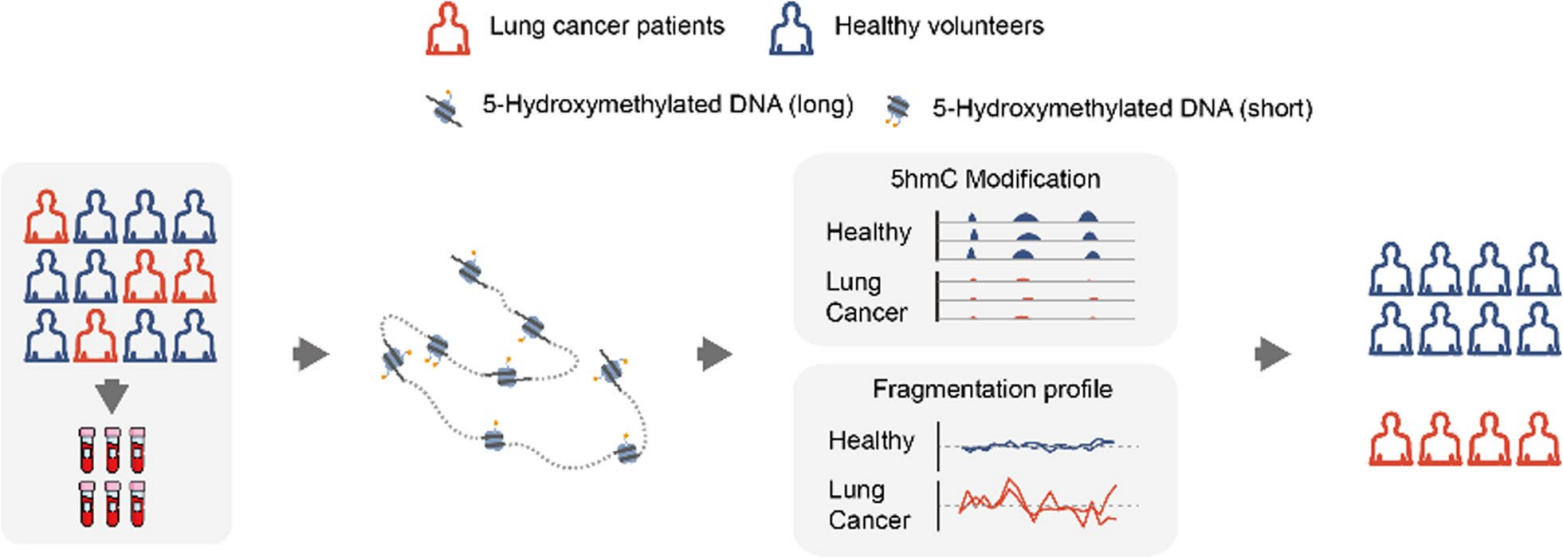

**Supplementary Information:**

The online version contains supplementary material available at 10.1186/s13148-022-01233-7.

## Background

Lung cancer is the most common cause of cancer death worldwide [1], causing approximately 1.6 million deaths annually, with a low five-year survival rate of approximately 15.9% due to difficult diagnosis at an early stage [2]. Small cell lung cancer (SCLC), a high-grade neuroendocrine carcinoma that accounts for 15% of all lung cancers, and non-small cell lung cancer (NSCLC), accounting for approximately 85% of lung cancers, are the two main subtypes [3]. Although low-dose computed tomography (LDCT) has been demonstrated to decrease mortality in high-risk groups in some trial research [4, 5], low specificity and radiation exposure have become the main reasons for underutilization [6, 7]. Several studies have investigated proteins, gene expression levels or microRNAs [8–12] as promising biomarkers in early detection, but few have been approved for routine clinical screening due to the high false-positive rate [2, 13]. Therefore, there is an urgent need for the development of noninvasive and sensitive approaches.

5-hydroxymethylation of cytosine (5hmC), which results from 5-methylcytosine (5mC) oxidation by the ten-eleven translocation proteins, is a novel epigenetic DNA modification [14]. Previous studies have revealed that 5hmC modification was enriched in gene bodies, promoters and enhancers, and was associated with gene expression levels [15, 16]. Aberrant methylation and hydroxymethylcytosine at the 5-position of cytosine are two main forms of epigenetic alterations contributing to tumor initiation and progression [17–20]. Abnormal 5hmC modification frequently appears in many solid tumors compared to corresponding normal tissues and could be identified as features of carcinogenesis [21]. The diagnostic and prognostic value of 5-hydroxymethylation of cytosine (5hmC) in cfDNA have been reported in several human cancers [16, 22–26], which highlighted the potential value of 5hmC in cancer diagnostics. Zhang et al. reported the characteristics of the genome-wide 5hmC signature and its diagnostic potential in NSCLC patients [23]. However the small sample size might limit the scope of related research. Also the genome-wide 5hmC profile of SCLC has not yet been reported. SCLC tends to grow and spread faster than NSCLC. Approximately, 70% of patients with SCLC [27] will have cancer that has already spread at the time they are diagnosed, with macrometastases commonly found in the lymph nodes, brain, liver, and bones. Thus, there is an urgent clinical need for the development of noninvasive approaches to improve SCLC early detection and ultimately the general population. And the potency and reliability of cell-free 5hmC as a diagnostic biomarker for SCLC remain elusive.

Plasma cfDNA is known to be highly fragmented [28]. Generally, cfDNA circulates in fragments ranging between 120 and 220 bp, commonly showing a prominent mode at 167 bp [29]. CfDNA fragments carrying tumor-mutated alleles were observed shorter than size of nucleosomal DNA (multiples of 167 bp) and size of cfDNA fragment with no tumor-mutated alleles [29, 30]. The pattern of different cfDNA fragment sizes could be defined as fragmentation, a feature with potential diagnostic power. Recent studies [29–31] depicted a large number of abnormalities in the cfDNA of cancer patients through genome-wide analysis of fragmentation patterns. Methods based on fragmentation, such as DELFI (DNA evaluation of fragments for early interception) [31], were developed to increase sensitivity of noninvasive detection of cancer, which to some extent could remedy shortcomings of methods based on mutations in circulating tumor DNA [32], i.e., individuals with cancer may be missed by targeted high coverage sequencing. However, in these researches, fragmentation profiles were produced from low coverage WGS. Investigation of the size distribution in 5hmC cfDNA datasets remains to be explored. We wondered whether fragmentation profiles were also available in 5hmC-enriched data and could serve as a diagnostic method to improve detection power of 5hmC cfDNA datasets.

In this study, we profiled 5hmC signatures and fragmentation patterns in cfDNA from a cohort of 157 lung cancer patients and 189 healthy individuals by using the 5hmC-Seal approach to investigate their diagnostic potential. Our results revealed that 5hmC features and size information obtained from 5hmC cfDNA data of lung cancer patients exhibited distinct patterns compared to those obtained from the cfDNA of the healthy controls. Using the elastic-net regression model, three sensitive and reliable models (5hmC-model, fragment-model and integrated-model) were trained for cancer detection, and all models performed well in an external validation set from Zhang et al. [23]. In particular, the integrated model remains stable in different stages and lung cancer subtypes. Corresponding genes of several 5hmC markers were found to be associated with overall survival (OS) and disease-free survival (DFS) in gene expression data from TCGA.

## Results

### Characteristics of samples and cfDNA 5hmC sequencing data

**Fig. 1:**
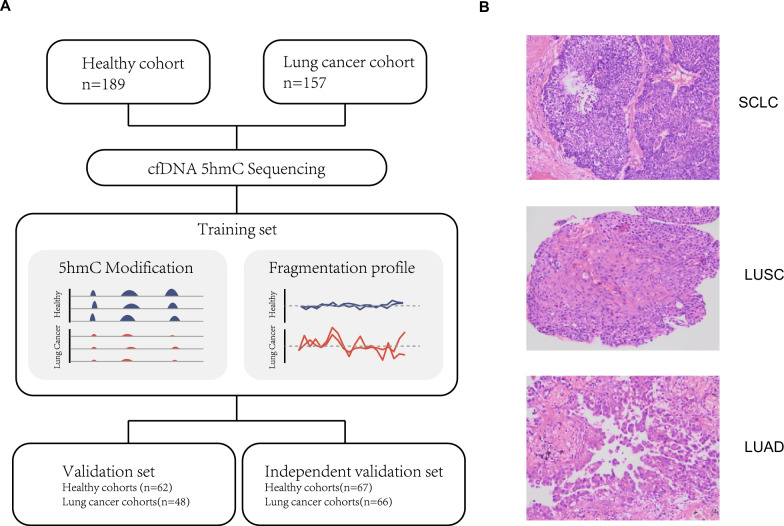
Overview of analysis pipeline and sample information. **A** The workflow chart of the study design. A total of 346 subjects, including 189 healthy volunteers and 157 lung cancer patients, were enrolled in analysis. 5hmC signals and fragmentation profiles were investigated in cfDNA 5hmC dataset. An integration model was finally constructed by combining cfDNA 5hmC features and fragmentation profiles for lung cancer diagnosis. **B** Representative images of hematoxylin and eosin (HE) staining in different histological types of lung cancers. Scale bar, 200 μm

To gain a comprehensive understanding of genome-wide 5hmC modifications and fragmentation related to lung cancer, we recruited 189 healthy individuals and 157 Chinese-descent lung cancer patients in our study (Fig. 1a, Table 1), which mainly included 62 lung adenocarcinoma (LUAD), 48 lung squamous cell carcinoma (LUSC), and 25 small cell lung cancer (SCLC) patients. Lung cancer cohorts were revealed by hematoxylin and eosin staining (Fig. 1b). A total of 83.44% were at advanced stages (TNM stages III 49 patients and IV 82 patients). Detailed information regarding subject characteristics is provided in Table 1.

**Table 1:** Clinicopathological characteristics of all the participants

	Lung cancer group (*N* = 157)	Healthy group (*N* = 189)	*P* value
Age, average (min, max)	62.3(37,80)	54.8(22,80)	< 0.0001
*Gender, n (%)*			< 0.0001
Male	106(67.5%)	55(29.1%)	
Female	51(32.5%)	118(62.4%)	
NA	0	16(8.5%)	
*TNM, n (%)*		/	
I	3(1.91%)		
II	9(5.73%)		
III	49(31.21%)		
IV	82(52.23%)		
NA	14(8.92%)		
*Histology*			
LUAD	62(39.49%)		
LUSC	48(30.57%)		
SCLC	25(15.92%)		
Others	22(14.01%)		

Clinicopathological characteristics of all the participants

5hmC-Seal data of circulating cell-free DNA from participants were obtained and qualified for analysis. We demonstrated the highly specific enrichment of 5hmC fragments in our data by showing an average capture efficiency of 97.43% over reads mapping to 5hmC spike-in DNA (Additional file 1: Figure S1A). The median final unique non-duplicate mapping rate of the libraries was 0.81, and the median total mapped read pairs was ~ 19 M (Additional file 2: Table S1).

To delineate the genome-wide distribution patterns of cfDNA 5hmC, we defined 201 bp peaks as previously described for each sample [33]. Significant differences were observed between peak numbers of lung cancer samples and healthy controls (*P* value = 1.50E−2, mean peak number: 295,284 peaks for lung cancer samples, 312,799 peaks for healthy samples, Additional file 1: Figure S1B). Metagene analysis of gene bodies (± 3 kb) and enhancers showed that normalized read density was higher in lung cancer samples than in healthy controls (Additional file 1: Figure S1C, D). The genome-wide analysis of 5hmC peaks showed that 5hmC exhibited a preference for enrichment on CDS, 5′UTRs, 3′ UTRs, exons, and promoters while depletion in intergenic regions, which is consistent with previous studies [16, 23] (Additional file 1: Figure S1E). The above results indicated the robustness of our methods and the nature of the 5hmC distribution in cfDNA.

### Prediction of lung cancer by 5hmC biomarkers in cfDNA

**Fig. 2:**
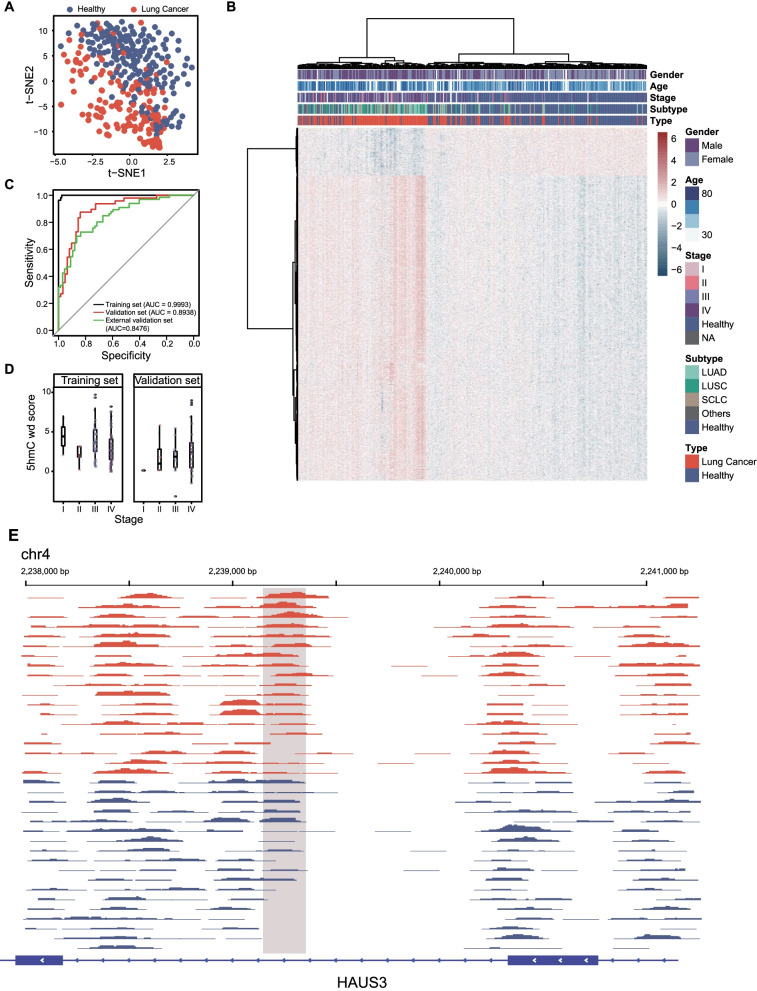
Cell-free 5hmC for detection of lung cancer. T-SNE plot (**A**) and heatmap (**B**) of 189 lung cancer patients and 157 healthy volunteers based on 3718 differentially hydroxymethylated peaks (DhMPs). Hierarchical clustering was performed across peaks and samples. **C** Performance of 5hmC model in the training set, validation set and public cfDNA 5hmC data retrieved from Zhang et al. containing 66 NSCLC patients and 67 healthy controls. **D** Boxplot of the wd-scores calculating with 5hmC model for stage I-IV lung cancer samples. **E** Genome Browser view of the 5hmC peaks in HAUS3 gene in chromosome 4 shows a marker locating within the gene (boxed region: chr4: 2239149–2239350). AUC, area under the curve; wd-score, weighted diagnosis score

Distinct cell-free 5hmC profiles were reported between NSCLC patients and healthy controls [23]. Most lung cancer samples could not be distinguished from healthy samples based on all 5hmC signal data (Additional file 1: Figure S2A). And subtypes of lung cancer samples could not be separated from each other (Additional file 1: Figure S2C). We further confirmed that batch effects were negligible, even after removing batch effects with limma [34] (Additional file 1: Figure S2B,D). To gain a global perspective of cell-free 5hmC differences between lung cancer patients at advanced tumor stages and healthy controls, we first compared 5hmC profiles from cfDNA of the two cohorts to identify differentially modified 5hmC loci. In total, 3718 differentially modified 5hmC loci were detected (*P* value < 0.001, adjusted *P* value < 0.05, absolute value of log_2_ fold change >  = 0.5), accounting for 0.3% of total loci (3718/974198), and these loci could separate most lung cancer patients from healthy controls (Fig. 2a, b). In order to associate biological functions to differentially modified 5hmC regions, we performed biological process enrichment analysis with GREAT (Genomic Regions Enrichment of Annotations Tool) [35]. GREAT enrichment analysis of these 3718 loci revealed 29 significantly enriched BP terms, among which endothelial cell fate commitment and negative regulation of DNA-dependent DNA replication were two representatives (Additional file 3: Table S2). Endothelial cells play a major role in the creation of supplemental blood vessels. This process is usually "hijacked" by cancer, which depends on neo-angiogenesis and vasculogenesis for growth and invasion [36]. Abnormal regulation of DNA replication also contribute to initiate uncontrolled growth of cancer cells [37]. The 5hmC changes of regions related to endothelial cell fate commitment and DNA replication might provide insights into lung cancer development. We then trained binomial regression models with elastic-net regularization through a fivefold cross-validation model selection scheme to develop a diagnostic model for distinguishing lung cancer patients from healthy controls using a randomly sampled training set consisting of 109 lung cancer patients and 127 healthy controls (Additional file 1: Figure S2C). With alpha = 0.2, our method selected 37 5hmC biomarkers with nonzero weight, which appeared in at least 4 crosses, to construct a diagnostic mode. It showed a good performance in the validation set (AUC = 0.8938, sensitivity = 87.5%, specificity = 83.87%), consisting of 48 lung cancer patients and 62 healthy controls. To further validate the robustness of our model, we utilized 5hmC data from NSCLC released by Zhang et al. [23] as an external validation set. The diagnostic performance was in agreement with our initial discovery cohort findings, and the AUC was 84.76% (sensitivity = 72.73%, specificity = 80.60%) (Fig. 2c). These results demonstrated and highlighted the reliability of our diagnostic model based on cfDNA 5hmC profiles. In addition, the 5hmC weighted diagnosis score (wd-score) computed based on the 5hmC diagnostic model showed no significant differences among lung cancer patients with variable tumor stages (Fig. 2d, Additional file 6: Table S5). The diagnostic model consisted of 37 5hmC loci that were distributed mostly in gene bodies except one in the promoter region (Additional file 4: Table S3). For example, a 5hmC marker with elevated signals in lung cancer patients was distributed in the gene body region of HAUS3 (Fig. 2e), a protein-coding gene that plays a key role in cytokinesis and mitosis.

### Fragmentation profiles from cfDNA 5hmC data for the detection of lung cancer

Usually, fragmentation profiles are based on low-coverage WGS of isolated cfDNA [30]. We speculated that the abnormal distribution of short-long cfDNA fragments could also occur in 5hmC-enriched cfDNA sequencing data, so the same method [30] was employed to explore the potential application of fragmentation profiles from cfDNA 5hmC data.

**Fig. 3:**
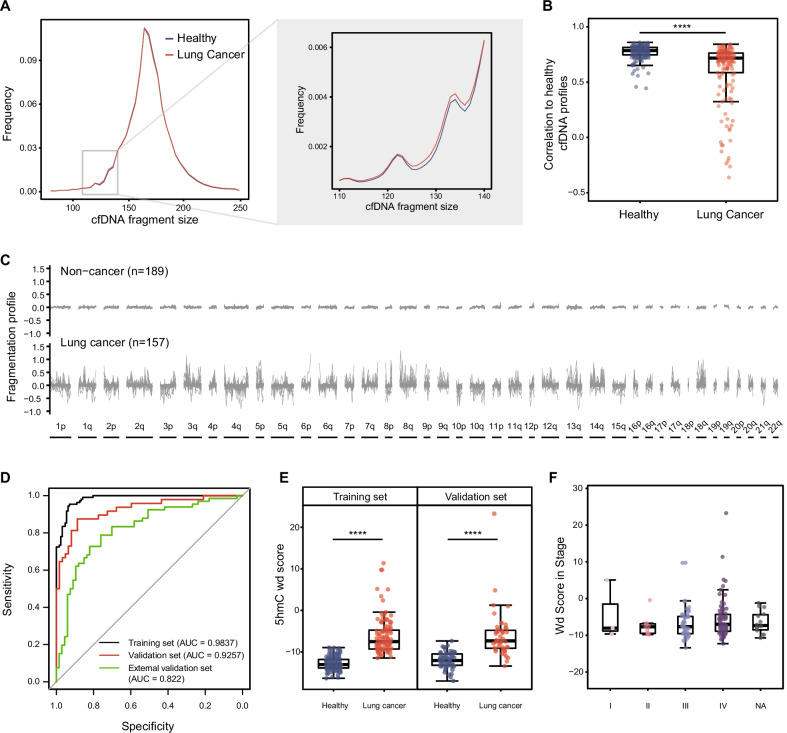
Fragmentation profiles from cfDNA 5hmC data for detection of lung cancer. **A** cfDNA fragment lengths distribution of healthy individuals (*n* = 189, grey) and patients with lung cancer (*n* = 157, blue) based on 4 bp windows. Patterns of 110 bp–140 bp based on 1 bp windows were shown in gray box. **B** Pearson correlations are depicted with box plots to show minimum, 25th percentile, median, 75th percentile, and maximum values. *P* value = 6.34E−19. **C** Genome-wide cfDNA fragmentation profiles (defined as the ratio of short to long fragments) from 346 cfDNA 5hmC data are shown in 5-Mb bins for 189 healthy individuals (top) and 157 patients with lung cancer (bottom). **D** ROC curves of fragmentation model for lung cancer patients and healthy controls in training set (black), validation set (red) and external validation set (green). **E** Boxplot of the fragment wd-score calculated with fragmentation model for lung cancer samples and healthy controls (training set *P* value = 1.52E−37, validation set *P* value = 2.29E−14). **F** Boxplot of the fragment wd-score calculated with fragmentation model for healthy controls, stage I lung cancer samples and stage II–IV lung cancer samples. *P < 0.05, **P < 0.01, ***P < 0.001, ****P < 1e − 5, Wilcoxon test

We first investigated fragment size distribution for both lung cancer patients and healthy controls. As expected [38, 39], there were more short fragments in lung cancer patients than in healthy samples (Fig. 3a), and the average lengths of fragments of 5hmC-enriched cfDNA from cancer patients were smaller than those from healthy individuals (168.4968 bp and 168.5556 bp, respectively). As previously described [31], quality-controlled mapped reads were analyzed in non-overlapping 5-megabase (Mb) windows to create genome-wide patterns. Within each window, we examined the short, long and total 5hmC-enriched cfDNA fragments and calculated the ratio of short to long fragments (RoSL). Then, Pearson correlation analyses were performed between each sample RoSL and the pseudo-healthy RoSL (median healthy RoSL). The results revealed that 5hmC-enriched cfDNA fragment profiles were consistent in healthy controls, while they were less stable in lung cancer patients (healthy samples mean correlation: 0.7699 lung cancer mean correlation: 0.6082, *P* value = 6.34e−19, Fig. 3b). Additionally, compared to healthy cfDNA profiles, lung cancer profiles had numbers of regions with increases and decreases in fragment sizes (Fig. 3c).

Based on the above results, GC-adjusted short and total 5hmC-enriched cfDNA fragment coverage of each 5 Mb window was utilized to construct an elastic net regression model to examine whether fragmentation profiles from 5hmC-enriched cfDNA have the ability to distinguish patients with cancer from healthy individuals (Additional file 1: Figure S3A). To exclude possible information from the validation set during model parameter training, the same training and validation groups from the 5hmC model were used. This approach suggested a set of 48 windows of fragmentation features with a weight of nonzero (alpha = 0.1), in which 17 windows utilized information of short fragments (Additional file 5: Table S4). The prediction model demonstrated acceptable accuracy in both the training set (sensitivity = 91.74%, specificity 93.70%, AUC = 0.9837) and the validation set (sensitivity = 87.50%, specificity = 80.65%, and AUC = 0.9257) (Fig. 3d). The public 5hmC data from Zhang et al. [23] were also included as an external validation set to validate the performance of the fragmentation model, which achieved results with AUC = 0.822 (sensitivity = 78.79%, specificity 76.12%, Fig. 3d).

Using the coefficients generated by the elastic net model, we obtained the wd-score (Additional file 6: Table S5), which could distinguish lung cancer patients from healthy controls (training set *P* value = 1.52E−37, validation set *P* value = 2.27E−14, Fig. 3e). Relationships between wd-score and cancer stages were also examined. However, no significant difference in wd-score was observed between different stages, despite stage IV possessing a higher median wd-score (Fig. 3f). There was no difference between lung cancer subtypes (Additional file 1: Figure S3B).

### Integration of 5hmC features and fragmentation profiles for improvement of lung cancer diagnosis

**Fig. 4:**
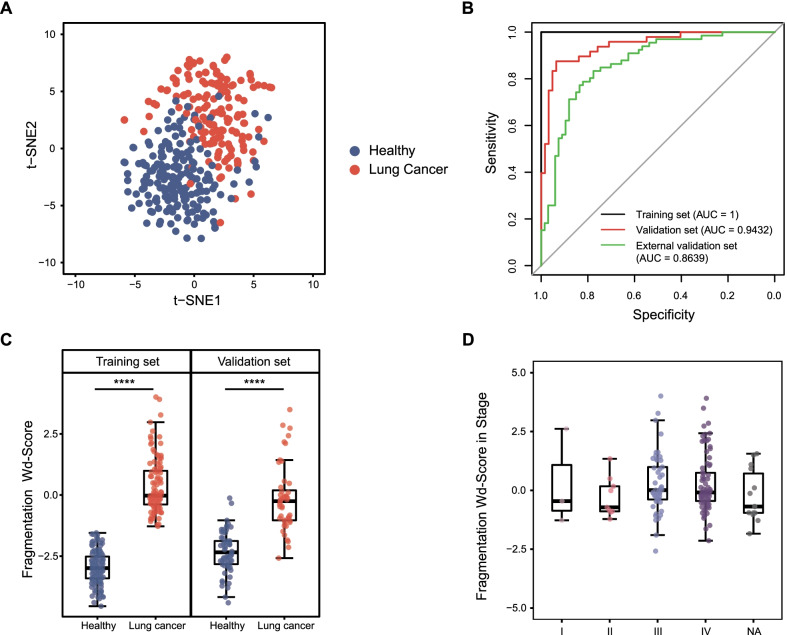
Performance of the 5hmC-fragmentation integrated model for lung cancer detection. **A** T-SNE plot of paired 5hmC features and fragmentation profiles data, based on the 85 features including the 37 5hmC biomarkers and 48 fragmentation areas. **B** Performance of the 5hmC-fragmentation integrated model in the training set, validation set and external validation set. **C** Boxplot of wd-score deriving from the integrated model for the lung cancer samples and the healthy controls (training set *P* value = 5.41E−40, validation set *P* value = 1.91E−15). **D** Boxplot of the wd-scores from the integrated model for stage I–IV lung cancer samples.*P < 0.05, **P < 0.01, ***P < 0.001, ****P < 1e−5, Wilcoxon test

5hmC modifications and fragmentation profiles from 5hmC cfDNA sequencing data characterized two natures of cfDNA. We utilized these two types of features to construct an integrated model containing 37 5hmC markers and 48 fragmentation markers. t-SNE analysis revealed that the combination can discriminate lung cancers from healthy controls (Fig. 4a). Then, elastic-net regression with tenfold cross-validation was performed to build the integrated model, which achieved AUCs of 1, 0.9432, and 0.8639 in the training set, validation set and external validation set, respectively (Fig. 4b). A better diagnostic efficiency was achieved by this co-modeling method. We also checked prediction power in different stages. In our validation set, stage I–II disease (*n* = 5), stage III disease (*n* = 12) and stage IV disease (*n* = 27) all had good performances (AUC_I–II_ = 0.9677, AUC_III_ = 0.9126, AUC_IV_ = 0.9564, respectively, Additional file 1: Figure S4A-C). Analyses of different histologic subtypes of lung cancer in the validation set showed that small cell lung cancer (SCLC, *n* = 8) and lung adenocarcinoma (LUAD, *n* = 18) were more easily detected than lung squamous cell cancer (LUSC, *n* = 13) (AUC_LUAD_ = 0.9615, AUC_LUSC_ = 0.9057, AUC_SCLC_ = 0.9899, Additional file 1: Figure S4D-F).

The Wd-score derived from the new model indicated a significant difference between healthy controls and lung cancer patients (training set *P* value = 5.41E−40, validation set *P* value = 1.91E−15, Fig. 4c). However, there was no significant difference between stages (Fig. 4d) or histologic subtypes (Additional file 1: Figure S4G).

### 5hmC markers signify genes associated with OS and DFS

**Fig. 5:**
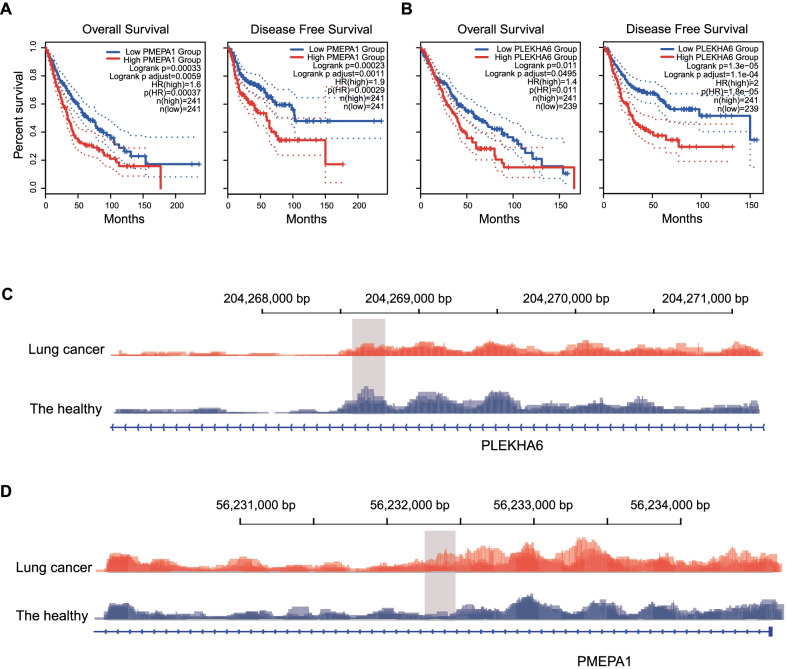
Genes marked by 37 diagnostic 5hmC biomarkers associated with lung cancer survival. Kaplan–Meier curves of overall survival and disease free survival in LUAD-LUAC cohorts from TCGA based on gene expression of PMEPA1 (**A**) and PLEKHA6 (**B**). Stacked genome browser plot of the 5hmC peaks located in promoter or gene body region of PLEKHA6 (**C**) and PMEPA1 (**D**)

In addition to distinguishing lung cancer patients from healthy controls, we next determined the performance of our 5hmC markers for their ability to stratify lung cancer patients by the survival time of overall survival (OS) and disease-free survival (DFS). The final 37 5hmC loci in the diagnostic model were first assigned to genes to obtain 18 5hmC-associated genes. Then, gene expression data of those 18 genes in the TCGA cohort were utilized to estimate the prognostic value of each gene in LUAD group, LUSC group and LUAD-LUSC group, respectively. Interestingly, the mRNA expression of both PLEKHA6 and PMEPA1 showed a significant correlation with both OS and DFS in LUAD-LUSC group. Patients with low PMEPA1 expression values exhibited longer OS and DFS in LUAD-LUSC group (OS: HR = 1.6, adjusted *P* value = 0.0059; DFS: HR = 1.9, adjusted *P* value = 0.0011; Fig. 5a). But in the two main subtypes of lung cancer, LUAD and LUSC, this group only showed longer OS in LUAD group (HR = 1.6, adjusted *P* value = 0.019) and longer DFS in LUSC group (HR = 1.6, adjusted *P* value = 0.0384, Additional file 1: Figure S5A). PMEPA1 is a transmembrane protein that was originally identified as a prostatic RNA, alternatively termed TMEPAI. It is identified as a direct target gene of transforming growth factor-β (TGF-β)/Smad signaling that participates in negative feedback control of the duration and intensity of TGF-β/Smad signaling [40]. Studies showed that highly expressed PMEPA1 suppressed levels of Smad phosphorylation in lung cancer cells and reduced the growth inhibitory effects of TGF-β/Smad signaling to enhance tumorigenic activities in lung cancer cells [41], which indicated possible poor prognosis in PMEPA1 highly expressed group.

Compared with patients with low PLEKHA6 expression, patients with high PLEKHA6 expression showed shorter OS and DFS in LUAD-LUSC group (OS: HR = 1.4, adjusted *P* value = 0.00495; DFS: HR = 2, adjusted *P* value = 0.00011; Fig. 5b). In LUAD group, patients with high PLEKHA6 expression had shorter OS (HR = 1.7, adjusted *P* value = 0.0054) In LUSC group, patients with high PLEKHA6 expression exhibited shorter OS and DFS (OS: HR = 1.8, adjusted *P* value = 0.0342; DFS: HR = 2.1, adjusted *P* value = 0.0384; Additional file 1: Figure S5B). The biological mechanism between PLEKHA6 and lung cancer still remained unclear. But it has been reported that PLEKHA6 was another novel survival predictor, which was associated with breast cancer mortality [42]. We also assigned 3718 differentially hydroxymethylated peaks (DhMPs) to 1378 genes. We used GEPIA2 to generate OS survival plots in LUAD group, LUSC group and LUAD-LUSC group, respectively. All the plots showed no correlation between OS/DFS and the 1378 signatures group (Additional file 1: Figure S5C,D). Using Integrative Genomics Viewer [43], genome browser plots of the 5hmC locus located in PLEKHA6 and PMEPA1 were illustrated in both lung cancer patients and healthy controls (Fig. 5c, d).

## Discussion

Liquid biopsy has been proved to be an effective and sensitive method for cancer detection [16, 44]. Recent studies have reported that 5hmC, an important component of the mammalian genome [45], plays an important role in gene expression regulation and carcinogenesis [21]. These features indicate the potential value of 5hmC in cancer diagnostics [46]. Nano-hmC-Seal and hMe-Seal were invented to utilize this sensitive characteristic to distinguish different patient conditions [16, 22].

Our research recruited 157 lung cancer samples and 189 healthy controls, which constituted the largest lung cancer cfDNA 5hmC dataset to date, especially with 25 SCLC samples. The median survival for SCLC patients is only 7–12 months after diagnosis, which reveals the urgency of diagnostic methods for early detection of SCLC [47]. We utilized a cost-effective and sensitive 5hmC-Seal method [16] to study profiles of 5hmC and fragmentation embedded in 5hmC cfDNA datasets. Then, we constructed diagnostic models based on 5hmC signals, fragmentation profiles, and features integrating 5hmC signals and fragmentation profiles with acceptable accuracy, not only discriminating lung cancer patients from healthy controls but also performing well in different stages and cancer subtypes. Corresponding genes of some 5hmC markers were found to be associated with DFS and OS, which might provide insights into disease management and prognosis. Also DhMP related gene PLEKHA6 might provide an interesting insight into biological mechanism of lung cancer. Overall, our findings determined that epigenetic signatures and fragmentation profiles in cfDNA 5hmC datasets had the potential to serve as biomarkers for NSCLC and SCLC.

Our results consisting of higher 5hmC levels in the gene body (± 3 kb) in lung cancer patients compared to healthy controls (Additional file 1: Figure S1C) are consistent with a previous study reported by Zhang et al. but inconsistent with results from Song et al., implying that ethnic differences might result in diverse cancer profiles in different populations [48]. Thus, different ethnic groups and even geographical groups could be included to inquire about common features to make liquid biopsies extensively available.

In 5hmC model construction, a 201 bp fixed-width peak method was utilized, as it focused more on 5hmC-enriched regions and reduced the risks of finding biomarkers without biological meanings, as methods such as gene bodies and sliding windows might lead to statistically significant differences. Further, short peaks could bring this noninvasive method into clinical practice more easily.

Along with rapid technological and analytical advancements in recent years, fragment patterns of cfDNA in peripheral blood have provided new insights into noninvasive detection. Analyses of fragmentation profiles in cfDNA permit evaluation of natural size distribution and genomic or chromatin characteristics across the whole genome except for epigenetic modifications. All studies of fragmentation were based on low-coverage WGS till now. The fragmentation profiles from 5hmC cfDNA datasets remain poorly characterized. Therefore, we adopted the same methods as DELFI in 5hmC cfDNA sequencing data to examine whether fragment size information is available in 5hmC-enriched data. The results indicated that RoSL was still present after enrichment, and the fragmentation model achieved an AUC of 0.822. We then explored the combination of epigenetic modifications and fragmentation profiles, and the results suggested that conjoint analysis improved detection power. Taken together, these findings indicated that cfDNA 5hmC sequencing may provide a promising noninvasive approach for cancer diagnosis and prognosis.

In clinical applications, digital criteria such as CA199 might be more acceptable and preferred for doctors and patients. In this way, the wd-score was calculated using 5hmC or fragmentation profiles with coefficients of the elastic-net regression model. The results revealed that there were different score distributions, which made it possible to achieve clinical transformation. However, we did not obtain associations between wd-score and stages or cancer subtypes, which might be due to insufficient samples.

The shortcomings of our research are obvious. First, more samples with different stages and large-scale subtypes are needed, especially early stages I and II, to make the robustness and potential value of our methods more convincing. Second, the lack of clinical information in our dataset, such as smoking history, alcohol history, chronic disease, and survival data, restricted us from associating cfDNA markers with clinicopathological characteristics, i.e., correlation of wd-score and tumor size, which might encourage us to explore the mechanisms of early tumor progression through 5hmC modifications. We are dedicated to collecting large-scale samples for further research and transforming them for clinical applications. In the future, we will adopt this approach for the diagnosis of malignant and benign nodules.

## Conclusions

The clinical significance of cfDNA 5hmC profiles from advanced lung cancer patients was demonstrated in our large cohorts consisting of both NSCLC and SCLC patients. Thirty-seven 5hmC markers were identified for detecting lung cancer, and for the first time, fragmentation profiles of cfDNA 5hmC were introduced into the diagnostic model, with 48 fragmentation features confirmed to have diagnostic efficiency. Moreover, the integrated model with the 5hmC signal and fragmentation profiles demonstrated increased detection power compared with models based on either of them. Taken together, this study provides a new perspective for clinical prediction models based on cfDNA 5hmC data, which are valuable in the clinical application of liquid biopsies for lung cancer diagnosis. More samples from different stages and subtypes are worthy of further investigation.

## Methods

### Study design

In total, 157 lung cancer patients and 189 healthy controls were retrospectively recruited to investigate the diagnostic potential value of cfDNA 5hmC and fragmentation biomarkers for lung cancer, which including 62 LUAD, 48 LUSC,25 SCLC and 22 other lung cancers. All subjects were recruited in a West China Hospital Institutional Review Board-approved protocol with informed consent. Lung cancer samples were confirmed histopathologically with no chemotherapy or radiotherapy for malignant tumors and healthy participants were enrolled from the community, which had no previous history for cancer. Cancer stages were classified according to the Eighth Edition Lung Cancer Stage Classification in AJCC/UICC cancer staging manuals [49].

### Blood sample processing, 5hmC library construction and sequencing

5hmC library construction was performed as previously described [16]. Firstly, 2-mL plasma samples were used to extract cfDNA using QIAamp Circulating Nucleic Acid Kit. Then, cfDNA (5–10 ng) ligated with sequencing adaptors was incubated in a 25 μL reaction solution containing HEPES buffer (50 mM, pH 8.0), 25 mM MgCl_2_, 60 μM N3-UDP-Glc (ActiveMotif, Carlsbad, CA, USA), and 12.5 U β-glucosyltransferase (NEB, Beverly, MA, USA) for 2 h at 37 °C. Next, 2.5ul DBCO-PEG4-biotin (Sigma, Carlsbad, CA, USA) was directly added and incubated for 2 h at 37 °C. 10 μg sheared salmon sperm DNA (Life Technologies, USA) was added before the Micro Bio-Spin 30 Column (Bio-Rad, Hercules, CA, USA) was used to purify the DNA following the instruction and then adjust the final volume to 25μL. After that, the purified DNA was incubated with 5-μL C1 streptavidin beads (Life Technologies, USA) in buffer 1 (5 mM Tris pH 7.5, 0.5 mM EDTA, 1 M NaCl and 0.2% Tween 20) for 30 min. The beads were subsequently undergone three 5-min washes each with buffer 1, buffer 2 (buffer 1 without NaCl), buffer 3 (buffer 1 with pH 9) and buffer 4 (buffer 3 without NaCl). Then, the beads were resuspended in water and amplified with 11 cycles of PCR amplification (initial denaturing at 98 °C for 45 s, followed by 11 cycles of denaturing at 98 °C for 15 s, annealing at 60 °C for 30 s, extension at 72 °C for 30 s, and a final extension at 17 °C for 5 min). The amplified products were purified using 0.8 × AMPure XP beads (Beckman Coulter, Fullerton, CA, USA). Pair-end 150 bp sequencing was performed on the Illumina Novaseq 6000 platform.

### cfDNA sequencing data processing

As the 5hmC-captured library was proceeded using 150-bp paired-end runs, the following methods were applied for 5hmC data alignment and 5hmC-based model. FastQC (version 0.11.8) was used to check data quality of 150 bp FASTQ files. Adapters of 150 bp FASTQ files were removed by Trimmomatic (version 0.38) [50]. Bowtie2 (version 2.3.4.3) [51] was used to process sequencing reads, which were aligned to hg19 and spike-in DNA with default parameters. Samtools (version 1.9) [52] was used to filter the generated SAM files with parameter settings of ‘-f 2 -F 1548 -q 30’ to include high quality, properly paired reads, followed by converting to BAM format. Picard (version 2.18.23) was employed to sort and index filtered SAM files and to ensure the removal of duplicate reads before subsequent analysis. Three types of spike-in DNA sequences were included into reference; then, capture efficiency, as a quality control measurement for the 5hmC and 5mC, was calculated as counts of reads aligned to a type-specific spike-in DNA divided by counts of reads aligned to total spike-in DNA.

Based on previous results of shorter cfDNA from cancer patients, we also gained raw FASTQ data (which include reads shorter than 150 bp paired-end reads). Adapters of raw FASTQ files were removed by cutadapt (version 1.9.1) and mapping was proceeded with bwa mem (version 0.7.5a-r405) [53] with default parameters. Samtools was utilized to obtain high-quality reads with ‘-f 2 -q 20’ and to remove PCR duplicate reads.

### Peak detection

Peak detection of 5hmC sequencing data followed steps as previously described [33]. Firstly, MACS2 (version 2.1.2) [54] was utilized to call peaks for each sequencing dataset. Secondly, in each sample, raw peak list generated by MACS2 was extended 100 bp on either side of the peak summits and peak scores were normalized to “score per million,” following by removing overlapping peaks. Thirdly, reproducible peaks in at least 10% of lung cancer samples or healthy controls with score per million >  = 5 were merged into group-specific (cancer-specific or healthy-specific) peak lists by removing overlapping peaks to generate the final consensus peak list.

All the peaks involved in the ENCODE hg19 blacklist, peaks that extend beyond any ends of chromosomes and peaks on chromosomes X, Y or on the mitochondrial genome, were filtered.

### 5hmC biomarkers selection and model construction

We firstly divided all cfDNA samples into two groups with a proportion of 4:1, the training set and the validation set (training set: 109 lung cancer samples and 127 healthy controls, validation set: 48 lung cancer samples and 62 healthy controls). Then, we determined differentially 5hmC loci for model construction by the following strategy: (a) randomly separating the training set into fivefolds and performed fivefold cross-validation, (b) At each cross, fourfolds were selected as cross-training set, and then 100 times repeats were performed to further select markers appeared in at least 95% iterations using elastic net model [55]. The final markers observed in at least one cross were used to build final prediction model in the training set and make predictions in the validation set. The α and λ was selected to maximize ROC in the training set over a grid of values (α range: 0.05–1 with 0.05 increment; λ range: 10–5–1 with logarithmically equal increment).

The final diagnostic model was identified as the one with best performance in the validation set. To verify the reliability of the final diagnostic model, we downloaded cfDNA 5hmC data of non-small cell lung cancer (NSCLC) from the Genome Sequence Archive in BIG Data Center with accession number PRJCA000816 (66 lung cancer samples and 67 healthy controls) as external validation set. The wd-score was calculated with coefficients of corresponding markers as follows:

$$Wd{-}score \, = \, sum \, \left( {coef \, \left( k \right) \, * \, FPKM \, \left( k \right)} \right),{\text{ where k represents the marker}}$$

### Fragmentation profile-based model construction

Raw fastq files with 189 healthy samples and 157 lung cancer samples were included in fragmentation analysis. As previously described [30], the hg19 autosomes were divided into 26,236 non-overlapping 100-kb bins, which excluding low mappability regions [56] and Duke blacklisted regions (http://hgdownload.cse.ucsc.edu/goldenpath/hg19/encodeDCC/wgEncodeMapability/). Short fragments were defined as having lengths between 80 and 150 bp and long fragments between 151 and 250 bp. Normalization was conducted as previously described [30]. In short, locally weighted scatterplot smoothing regression analysis (LOWESS) was employed to account for GC biases separately for short and long fragments in every single sample. Then total GC-adjusted coverage of 5-Mb windows were calculated to reduce possible noise.

In order to perform marker selection and establish lung cancer prediction models, the elastic net regularization on a logistic linear regression model was chosen, which implemented in the glmnet R package (version 2.0-18) [55]. The following procedure was applied:

We used the same training set and validation set as 5hmC model construction (training set: 109 lung cancer samples and 127 healthy controls, validation set: 48 lung cancer samples and 62 healthy controls, external validation set: 66 lung cancer samples and 67 healthy controls). GC-corrected total and short fragment coverage for all 504 bins were centered and scaled for each sample. To avoid overfitting, the training set was randomly divided into fivefolds and performed fivefold cross-validation. At each cross, fourfolds were selected as cross-training set then 100 times repeats were performed to further select markers appeared in at least 95% iterations using elastic net model. The final markers observed in at least five cross were used to build final prediction model in the training set and make predictions in the validation set. The α and λ were selected to maximize ROC in the training set over a grid of values (α range: 0.05–1 with 0.05 increment; λ range: 10^−5^–1 with logarithmically equal increment).

Genome-wide fragmentation profiles were generated using the ratio of short to long 5hmC-enriched cfDNA fragments using 5-Mb windows for each sample. The median healthy profile was calculated as the median ratio of short to long 5hmC-enriched cfDNA fragments in 5-Mb windows. Pearson correlation was calculated between individual profiles and median healthy profile.

### Integration of 5hmC and fragmentation model

In order to integrate 5hmC profiles and fragmentation profiles, the 37 5hmC markers and 48 fragmentation features were combined for elastic net model training, with 109 lung cancer samples and 127 healthy controls in the training set as well as 48 lung cancer and 62 healthy controls in the validation set. A grid of α from 0 to 1 and lambda from 10–5 to 1 with tenfold cross-validation were attempted to maximize ROC in the training set to confirm best parameters.

### Survival analysis

The final 37 diagnostic 5hmC loci were firstly mapped to gene regulatory regions (defined as 2500 bp upstream of the TSS and gene body regions) to get their associated genes. Actually, we get 21 associated genes that 37 5hmC loci marks. We further explored the prognostic value of those genes by seeking the relationship between gene expression level and survival data in GEPIA2 [57]. Gene expression thresholds for classifying patients into two groups (low and high) were defined as median or 75th quantile of the corresponding gene.

### Peak annotation and metagene analysis

BEDtools (version 2.25.0) [58] was used to get occupancy of peaks with each genomic element (> 1 bp), following by enrichment analysis assessed by odds ratio. Ngs.plot [59] was used to depict metagene profiles on gene body (± 3 kb) and enhancer. Gene Ontology (GO) analysis was performed by GREAT (version 4.0.4) [35].

### Statistical analysis

Statistical analyses were performed in R 3.6.3 environment. The Wilcoxon test was used to compare wd-score of different groups, with two-sided and *P* values < 0.05. The R packages RtSNE (version 0.15) [60] and pheatmap (version 1.0.12) were used for dimension reduction and clustering analysis. The glmnet (version 2.0-18) and caret (version 6.0-86) [61] package were utilized to construct models and select parameters. The pROC (version 1.15.3) [62] was used to generate receiver operating characteristic (ROC) curves and calculate the AUC.

## Supplementary Information

**Additional file 1**: **Figure S1**: **A** The 5hmC spike-in DNA is specifically enriched in the 5hmC libraries. Error bars indicate Standard Deviation (SD). **B** 5hmC peak number distribution of lung cancer samples and healthy samples. **C** Metagene profiles of the regions from TSS to TES with flanking 3 kb area. **D** Metagene profiles of enhancer with flanking 3kb area. **E** Enrichment analysis of 5hmC peaks of lung cancer patients and healthy controls overlapping with distinct genomic elements. TSS, transcription start sites; TES, transcription end sites. CDS, Coding DNA Sequence; 3′UTR, 3′untranslated region; 5′UTR, 5′untranslated region. **Figure S2**: **A** T-SNE analysis of cfDNA 5hmC data from lung cancer and healthy samples. **B** T-SNE plot of cfDNA 5hmC data from lung cancer and healthy samples in distinct batches. **C** T-SNE analysis of cfDNA 5hmC data from subtypes of lung cancer and healthy samples **D** T-SNE plot of cfDNA 5hmC data from lung cancer and healthy samples in distinct batches after removing batch effects. **E** Flow chart of 5hmC model construction. **Figure S3**: **A** Flow chart of fragmentation model construction. **B** Boxplot of the wd-scores from the integrated model for histologic subtypes of lung cancer samples. **Figure S4**: **A** Performance of integrated model in forms of receiver operating characteristic (ROC) curves and area under curve (AUC) scores in stage I-II, III (**B**), IV (**C**). **D** Performance of integrated model in forms of ROC curves and AUC scores in LUAD, LUSC (**E**), and SCLC (**F**). **G** Boxplot of the wd-scores from the integrated model for different histologic subtypes of lung cancer samples. **Figure S5**: Kaplan-Meier curves of overall survival and disease free survival in lung adenocarcinoma and lung squamous carcinoma from TCGA based on gene expression of PMEPA1 (**A**) and PLEKHA6 (**B**). **C**. Kaplan-Meier curves of overall survival of 1378 DhMP corresponding gene in LUAD+LUAC group, LUAD group and LUSC group. **D**. Kaplan-Meier curves of disease free survival of 1378 DhMP corresponding gene in LUAD+LUAC group, LUAD group and LUSC group.

**Additional file 2**: **Table S1**: Mapping summary of cfDNA 5hmC sequencing data.

**Additional file 3**: **Table S2**: GREAT enrichment analysis result of different 3718 loci from cfDNA 5hmC data.

**Additional file 4**: **Table S3**: List of 5hmC markers used in model construction.

**Additional file 5**: **Table S4**: List of fragmentation markers used in model construction.

**Additional file 6**: **Table S5**: Wd-scores of 189 healthy controls, 157 lung cancer patients and 133 external validation samples derived from distinct models.

## Data Availability

The datasets supporting the conclusions of this article are included within the article and its additional files. All other datasets used and analyzed during the study are available from the corresponding author on reasonable request.

## Abbreviations

5hmC: 5-Hydroxymethylcytosine
AJCC: American Joint Committee on Cancer
AUC: Area under the curve
cfDNA: Cell-free DNA
RoSL: Ratio of short to long fragments
IGV: Integrative genomics viewer
*t*SNE: T-distributed stochastic neighbor embedding analysis
wd-score: Weighted diagnosis score
LUAD: Lung adenocarcinoma
LUSC: Lung squamous cell carcinoma
SCLC: Small cell lung cancer

## References

[1] Siegel RL, Miller KD, Jemal A (2016). Cancer statistics, 2016. CA Cancer J Clin.

[2] Hoseok I, Cho JY (2015). Lung cancer biomarkers. Adv Clin Chem.

[3] Inamura K (2017). Lung cancer: understanding its molecular pathology and the 2015 WHO classification. Front Oncol.

[4] de Koning HJ (2020). Reduced lung-cancer mortality with volume CT screening in a randomized trial. N Engl J Med.

[5] National Lung Screening Trial Research, T., et al. Reduced lung-cancer mortality with low-dose computed tomographic screening*.* N Engl J Med. 2011;365(5):395–409. 10.1056/NEJMoa1102873 PMC4356534 21714641

[6] Richards TB, Soman A, Thomas CC (2020). Screening for lung cancer—10 states, 2017. MMWR Morb Mortal Wkly Rep.

[7] Nanavaty P, Alvarez MS, Alberts WM (2014). Lung cancer screening: advantages, controversies, and applications. Cancer Control.

[8] Chaturvedi AK (2010). C-reactive protein and risk of lung cancer. J Clin Oncol.

[9] Tang H (2018). Clinical significance of combined detection of interleukin-6 and tumour markers in lung cancer. Autoimmunity.

[10] Integrative Analysis of Lung Cancer, E., et al., *Assessment of lung cancer risk on the basis of a biomarker panel of circulating proteins.* JAMA Oncol, 2018. 4(10): e182078. 10.1001/jamaoncol.2018.2078 PMC6233784 30003238

[11] Silvestri GA (2015). A bronchial genomic classifier for the diagnostic evaluation of lung cancer. N Engl J Med.

[12] Seijo LM (2019). Biomarkers in lung cancer screening: achievements, promises, and challenges. J Thorac Oncol.

[13] Vargas AJ, Harris CC (2016). Biomarker development in the precision medicine era: lung cancer as a case study. Nat Rev Cancer.

[14] Tahiliani M (2009). Conversion of 5-methylcytosine to 5-hydroxymethylcytosine in mammalian DNA by MLL partner TET1. Science.

[15] Cui XL (2020). A human tissue map of 5-hydroxymethylcytosines exhibits tissue specificity through gene and enhancer modulation. Nat Commun.

[16] Song CX (2017). 5-Hydroxymethylcytosine signatures in cell-free DNA provide information about tumor types and stages. Cell Res.

[17] Ma K, Cao B, Guo M (2016). The detective, prognostic, and predictive value of DNA methylation in human esophageal squamous cell carcinoma. Clin Epigenet.

[18] Hao X (2017). DNA methylation markers for diagnosis and prognosis of common cancers. Proc Natl Acad Sci U S A.

[19] Pfeifer GP (2014). The role of 5-hydroxymethylcytosine in human cancer. Cell Tissue Res.

[20] Huang Y, Rao A (2014). Connections between TET proteins and aberrant DNA modification in cancer. Trends Genet.

[21] Jin SG (2011). 5-Hydroxymethylcytosine is strongly depleted in human cancers but its levels do not correlate with IDH1 mutations. Cancer Res.

[22] Li W (2017). 5-Hydroxymethylcytosine signatures in circulating cell-free DNA as diagnostic biomarkers for human cancers. Cell Res.

[23] Zhang J (2018). 5-Hydroxymethylome in circulating cell-free DNA as a potential biomarker for non-small-cell lung cancer. Genom Proteom Bioinform.

[24] Cao F (2020). Integrated epigenetic biomarkers in circulating cell-free DNA as a robust classifier for pancreatic cancer. Clin Epigenet.

[25] Cai, Z., et al., *Liquid biopsy by combining 5-hydroxymethylcytosine signatures of plasma cell-free DNA and protein biomarkers for diagnosis and prognosis of hepatocellular carcinoma.* ESMO Open. 2021;6(1):100021. 10.1016/j.esmoop.2020.100021 PMC7841321 33508734

[26] Tian X (2018). Circulating tumor DNA 5-hydroxymethylcytosine as a novel diagnostic biomarker for esophageal cancer. Cell Res.

[27] Ko J, Winslow MM, Sage J. Mechanisms of small cell lung cancer metastasis*.* EMBO Mol Med. 2021;13(1): e13122. 10.15252/emmm.202013122 PMC7799359 33296145

[28] Fleischhacker M (2011). Methods for isolation of cell-free plasma DNA strongly affect DNA yield. Clin Chim Acta.

[29] Mouliere, F., et al., *Enhanced detection of circulating tumor DNA by fragment size analysis.* Sci Transl Med. 2018;10(466). 10.1126/scitranslmed.aat4921 PMC6483061 30404863

[30] Cristiano S (2019). Genome-wide cell-free DNA fragmentation in patients with cancer. Nature.

[31] Mathios D (2021). Detection and characterization of lung cancer using cell-free DNA fragmentomes. Nat Commun.

[32] Phallen J et al., Direct detection of early-stage cancers using circulating tumor DNA. Sci Transl Med. 2017;9(403). 10.1126/scitranslmed.aan2415 PMC6714979 28814544

[33] Corces MR, et al. The chromatin accessibility landscape of primary human cancers. Science. 2018;362(6413). 10.1126/science.aav1898 PMC6408149 30361341

[34] Ritchie ME et al. limma powers differential expression analyses for RNA-sequencing and microarray studies*.* Nucl Acids Res. 2015;43(7):e47. 10.1093/nar/gkv007 PMC4402510 25605792

[35] McLean CY (2010). GREAT improves functional interpretation of cis-regulatory regions. Nat Biotechnol.

[36] Gu JW (2012). Notch signals in the endothelium and cancer "stem-like" cells: opportunities for cancer therapy. Vasc Cell.

[37] Vassilev A, DePamphilis ML. Links between DNA replication, stem cells and cancer*.* Genes (Basel). 2017;8(2). 10.3390/genes8020045 PMC5333035 28125050

[38] Jiang P (2015). Lengthening and shortening of plasma DNA in hepatocellular carcinoma patients. Proc Natl Acad Sci U S A.

[39] Snyder MW (2016). Cell-free DNA comprises an in vivo nucleosome footprint that informs its tissues-of-origin. Cell.

[40] Watanabe Y (2010). TMEPAI, a transmembrane TGF-beta-inducible protein, sequesters Smad proteins from active participation in TGF-beta signaling. Mol Cell.

[41] Vo Nguyen TT (2014). TMEPAI/PMEPA1 enhances tumorigenic activities in lung cancer cells. Cancer Sci.

[42] Aushev VN (2019). Tumor expression of environmental chemical-responsive genes and breast cancer mortality. Endocr Relat Cancer.

[43] Thorvaldsdottir H, Robinson JT, Mesirov JP (2013). Integrative Genomics Viewer (IGV): high-performance genomics data visualization and exploration. Brief Bioinform.

[44] Shen SY (2018). Sensitive tumour detection and classification using plasma cell-free DNA methylomes. Nature.

[45] Shen L (2014). Mechanism and function of oxidative reversal of DNA and RNA methylation. Annu Rev Biochem.

[46] Vasanthakumar A, Godley LA (2015). 5-hydroxymethylcytosine in cancer: significance in diagnosis and therapy. Cancer Genet.

[47] Chung HC (2020). Pembrolizumab after two or more lines of previous therapy in patients with recurrent or metastatic SCLC: results from the KEYNOTE-028 and KEYNOTE-158 studies. J Thorac Oncol.

[48] Campbell DE, Greenberg ER. Racial differences in the treatment of early-stage lung cancer*.* N Engl J Med. 2000;342(7):517; author reply 518–9. 10.1056/NEJM200002173420716 10691492

[49] Detterbeck FC et al. The eighth edition lung cancer stage classification*.* Chest. 2017;151(1):193–203. 10.1016/j.chest.2016.10.010 27780786

[50] Bolger AM, Lohse M, Usadel B (2014). Trimmomatic: a flexible trimmer for Illumina sequence data. Bioinformatics.

[51] Langmead B, Salzberg SL (2012). Fast gapped-read alignment with Bowtie 2. Nat Methods.

[52] Li H (2009). The sequence alignment/map format and SAMtools. Bioinformatics.

[53] Li H, Durbin R (2009). Fast and accurate short read alignment with Burrows-Wheeler transform. Bioinformatics.

[54] Zhang Y (2008). Model-based analysis of ChIP-Seq (MACS). Genome Biol.

[55] Friedman J, Hastie T, Tibshirani R (2010). Regularization paths for generalized linear models via coordinate descent. J Stat Softw.

[56] Fortin JP, Hansen KD (2015). Reconstructing A/B compartments as revealed by Hi-C using long-range correlations in epigenetic data. Genome Biol.

[57] Tang Z (2019). GEPIA2: an enhanced web server for large-scale expression profiling and interactive analysis. Nucl Acids Res.

[58] Quinlan AR, Hall IM (2010). BEDTools: a flexible suite of utilities for comparing genomic features. Bioinformatics.

[59] Shen L et al. ngs.plot: quick mining and visualization of next-generation sequencing data by integrating genomic databases*.* BMC Genom. 2014;15: 284. 10.1186/1471-2164-15-284 PMC4028082 24735413

[60] Van der Maaten L, Hinton G. Visualizing data using t-SNE*.* J Mach Learn Res. 2008;9(11).

[61] Kuhn M (2008). Building predictive models in R using the caret package. J Stat Softw.

[62] Robin X (2011). pROC: an open-source package for R and S+ to analyze and compare ROC curves. BMC Bioinform.

