# Supplementary material for: Integrated 5-hydroxymethylcytosine and fragmentation signatures as enhanced biomarkers in lung cancer
Source: Clin Epigenetics. 2022 Jan 24;14:15. doi: 10.1186/s13148-022-01233-7 (PMC8787948; doi:10.1186/s13148-022-01233-7)
Supplement: Supplementary file 1 — Additional file 1: Figure S1: A The 5hmC spike-in DNA is specifically enriched in the 5hmC libraries. Error bars indicate Standard Deviation (SD). B 5hmC peak number distribution of lung cancer samples and healthy samples. C Metagene profiles of the regions from TSS to TES with flanking 3 kb area. D Metagene profiles of enhancer with flanking 3kb area. E Enrichment analysis of 5hmC peaks of lung cancer patients and healthy controls overlapping with distinct genomic elements. TSS, transcription start sites; TES, transcription end sites. CDS, Coding DNA Sequence; 3′UTR, 3′untranslated region; 5′UTR, 5′untranslated region. Figure S2: A T-SNE analysis of cfDNA 5hmC data from lung cancer and healthy samples. B T-SNE plot of cfDNA 5hmC data from lung cancer and healthy samples in distinct batches. C T-SNE analysis of cfDNA 5hmC data from subtypes of lung cancer and healthy samples D T-SNE plot of cfDNA 5hmC data from lung cancer and healthy samples in distinct batches after removing batch effects. E Flow chart of 5hmC model construction. Figure S3: A Flow chart of fragmentation model construction. B Boxplot of the wd-scores from the integrated model for histologic subtypes of lung cancer samples. Figure S4: A Performance of integrated model in forms of receiver operating characteristic (ROC) curves and area under curve (AUC) scores in stage I-II, III (B), IV (C). D Performance of integrated model in forms of ROC curves and AUC scores in LUAD, LUSC (E), and SCLC (F). G Boxplot of the wd-scores from the integrated model for different histologic subtypes of lung cancer samples. Figure S5: Kaplan-Meier curves of overall survival and disease free survival in lung adenocarcinoma and lung squamous carcinoma from TCGA based on gene expression of PMEPA1 (A) and PLEKHA6 (B). C. Kaplan-Meier curves of overall survival of 1378 DhMP corresponding gene in LUAD+LUAC group, LUAD group and LUSC group. D. Kaplan-Meier curves of disease free survival of 1378 DhMP corresponding gene in LUAD+ [file 13148_2022_1233_MOESM1_ESM.pdf]

SupFig1

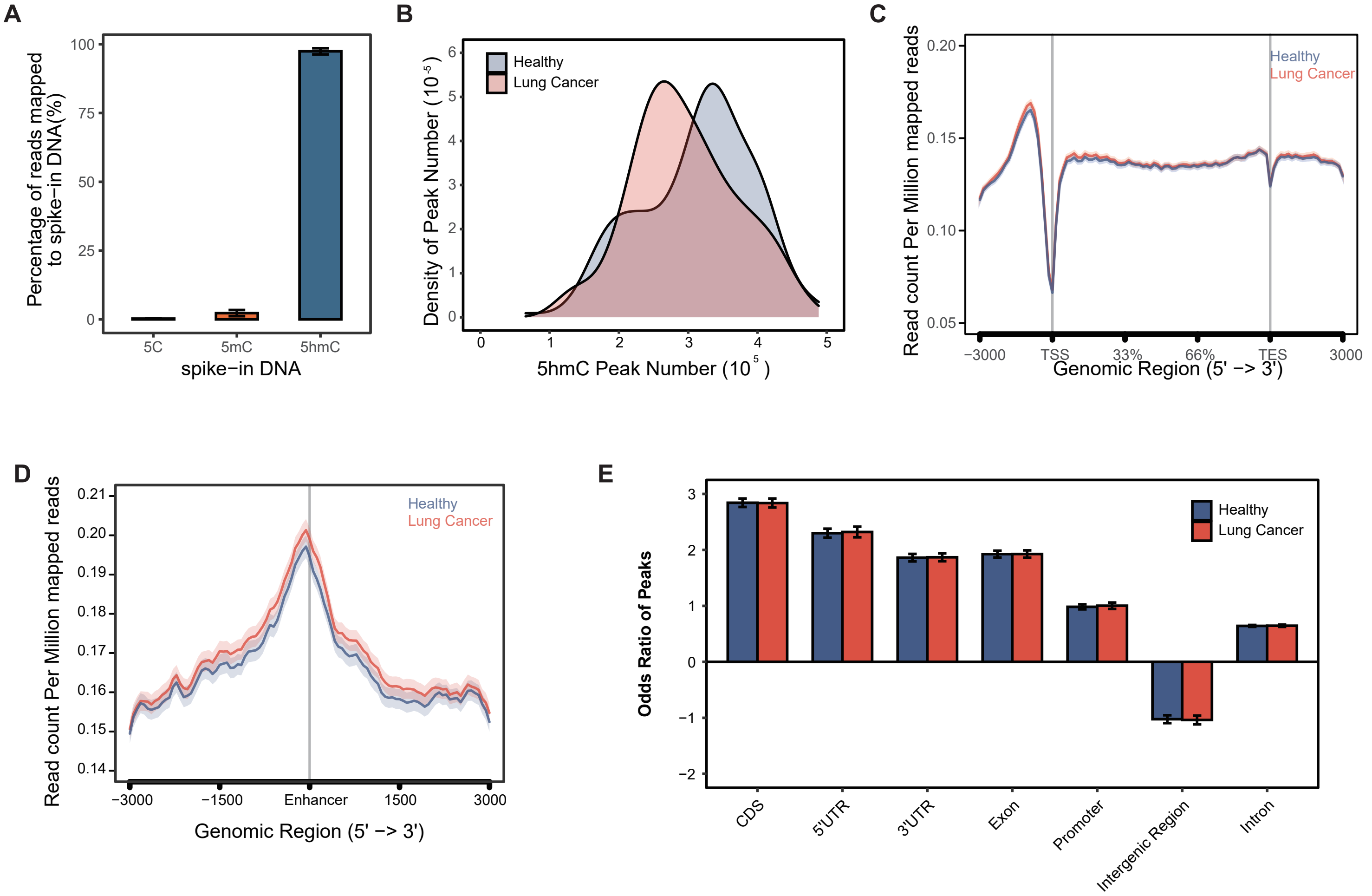

A
Percentage of reads mapped to spike−in DNA(%)
100
75
50
25
0
5C
5mC
5hmC
spike−in DNA
B
Healthy
Lung Cancer
Density of Peak Number (10⁻⁵)
5hmC Peak Number (10⁵)
C
Healthy
Lung Cancer
Read count Per Million mapped reads
0.20
0.15
0.10
0.05
−3000
TSS
33%
66%
TES
3000
Genomic Region (5' −> 3')
D
Healthy
Lung Cancer
Read count Per Million mapped reads
0.21
0.20
0.19
0.18
0.17
0.16
0.15
0.14
−3000
−1500
Enhancer
1500
3000
Genomic Region (5' −> 3')
E
Healthy
Lung Cancer
Odds Ratio of Peaks
3
2
1
0
−1
−2
CDS
5'UTR
3'UTR
Exon
Promoter
Intergenic Region
Intron

SupFig2

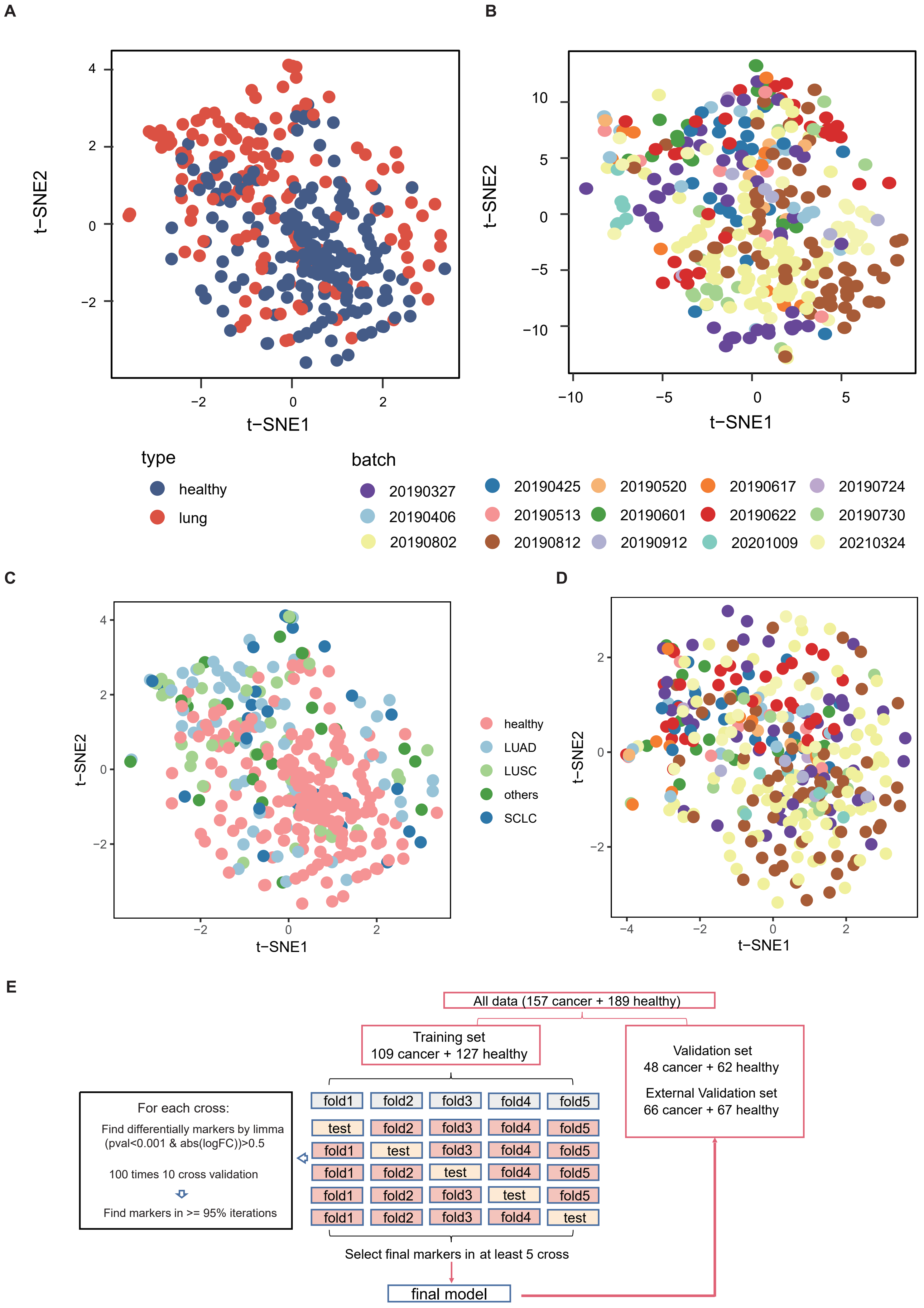
A
t−SNE2
t−SNE1
B
t−SNE2
t−SNE1
type
healthy
lung
batch
20190327
20190406
20190802
20190425
20190513
20190812
20190520
20190601
20190912
20190617
20190622
20201009
20190724
20190730
20210324
C
t−SNE2
t−SNE1
healthy
LUAD
LUSC
others
SCLC
D
t−SNE2
t−SNE1
E
All data (157 cancer + 189 healthy)
Training set
109 cancer + 127 healthy
Validation set
48 cancer + 62 healthy
External Validation set
66 cancer + 67 healthy
For each cross:
Find differentially markers by limma
(pval<0.001 & abs(logFC))>0.5
100 times 10 cross validation
Find markers in >= 95% iterations
fold1 fold2 fold3 fold4 fold5
test fold2 fold3 fold4 fold5
fold1 test fold3 fold4 fold5
fold1 fold2 test fold4 fold5
fold1 fold2 fold3 test fold5
fold1 fold2 fold3 fold4 test
Select final markers in at least 5 cross
final model

SupFig3

A

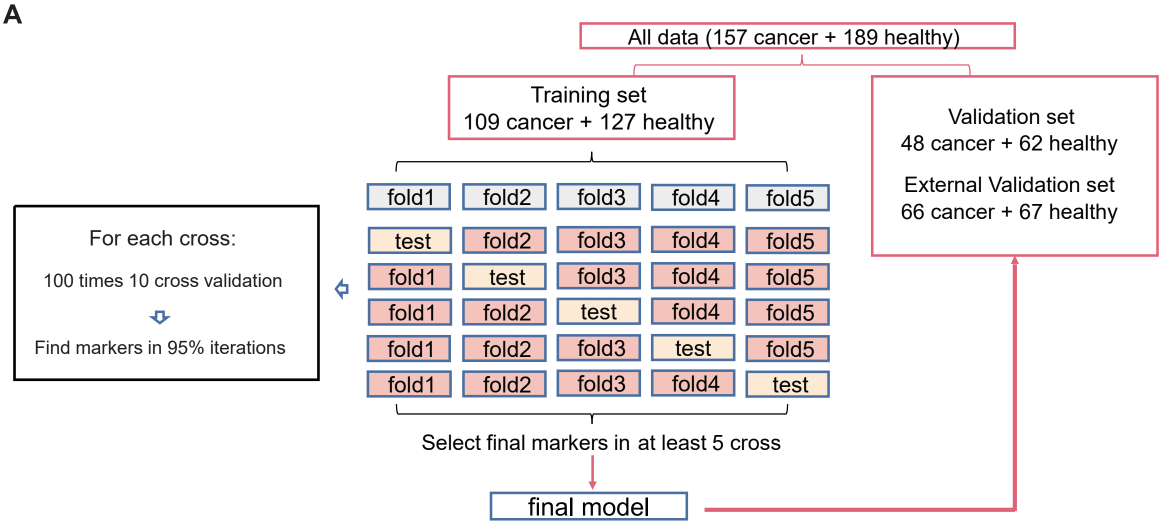
All data (157 cancer + 189 healthy)
Training set
109 cancer + 127 healthy
Validation set
48 cancer + 62 healthy
External Validation set
66 cancer + 67 healthy
fold1
fold2
fold3
fold4
fold5
test
For each cross:
100 times 10 cross validation
Find markers in 95% iterations
Select final markers in at least 5 cross
final model

B

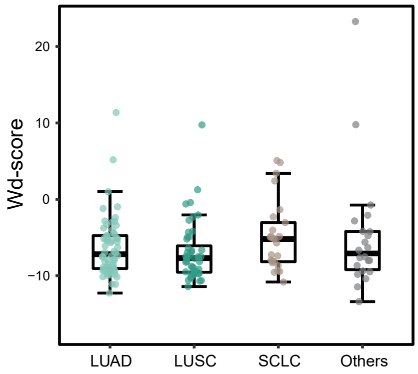
Wd-score
20
10
0
−10
LUAD
LUSC
SCLC
Others

SupFig4

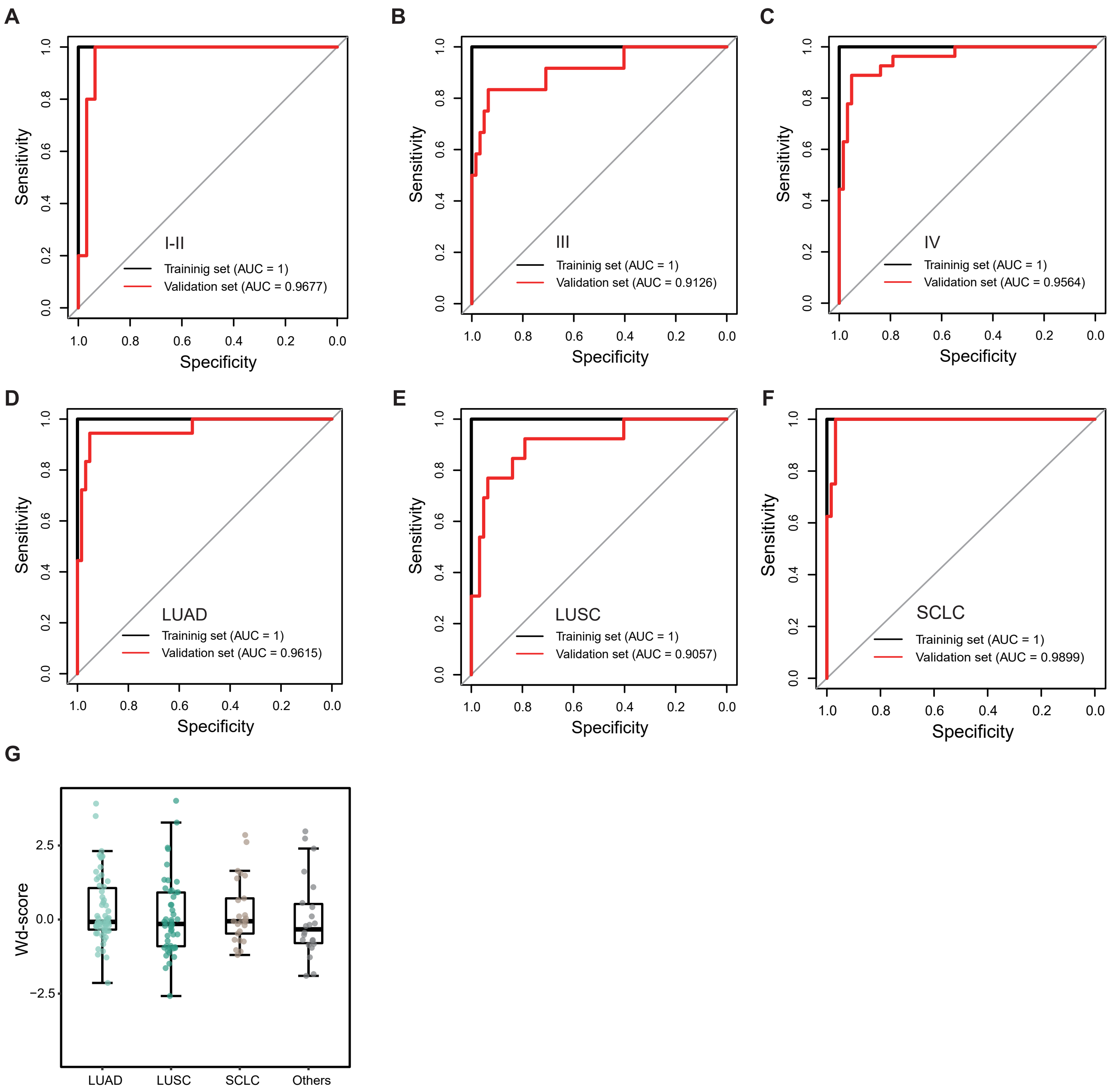
A
Sensitivity
Specificity
I-II
Traininig set (AUC = 1)
Validation set (AUC = 0.9677)
B
III
Traininig set (AUC = 1)
Validation set (AUC = 0.9126)
C
IV
Traininig set (AUC = 1)
Validation set (AUC = 0.9564)
D
LUAD
Traininig set (AUC = 1)
Validation set (AUC = 0.9615)
E
LUSC
Traininig set (AUC = 1)
Validation set (AUC = 0.9057)
F
SCLC
Traininig set (AUC = 1)
Validation set (AUC = 0.9899)
G
Wd-score
LUAD
LUSC
SCLC
Others

SupFig 5

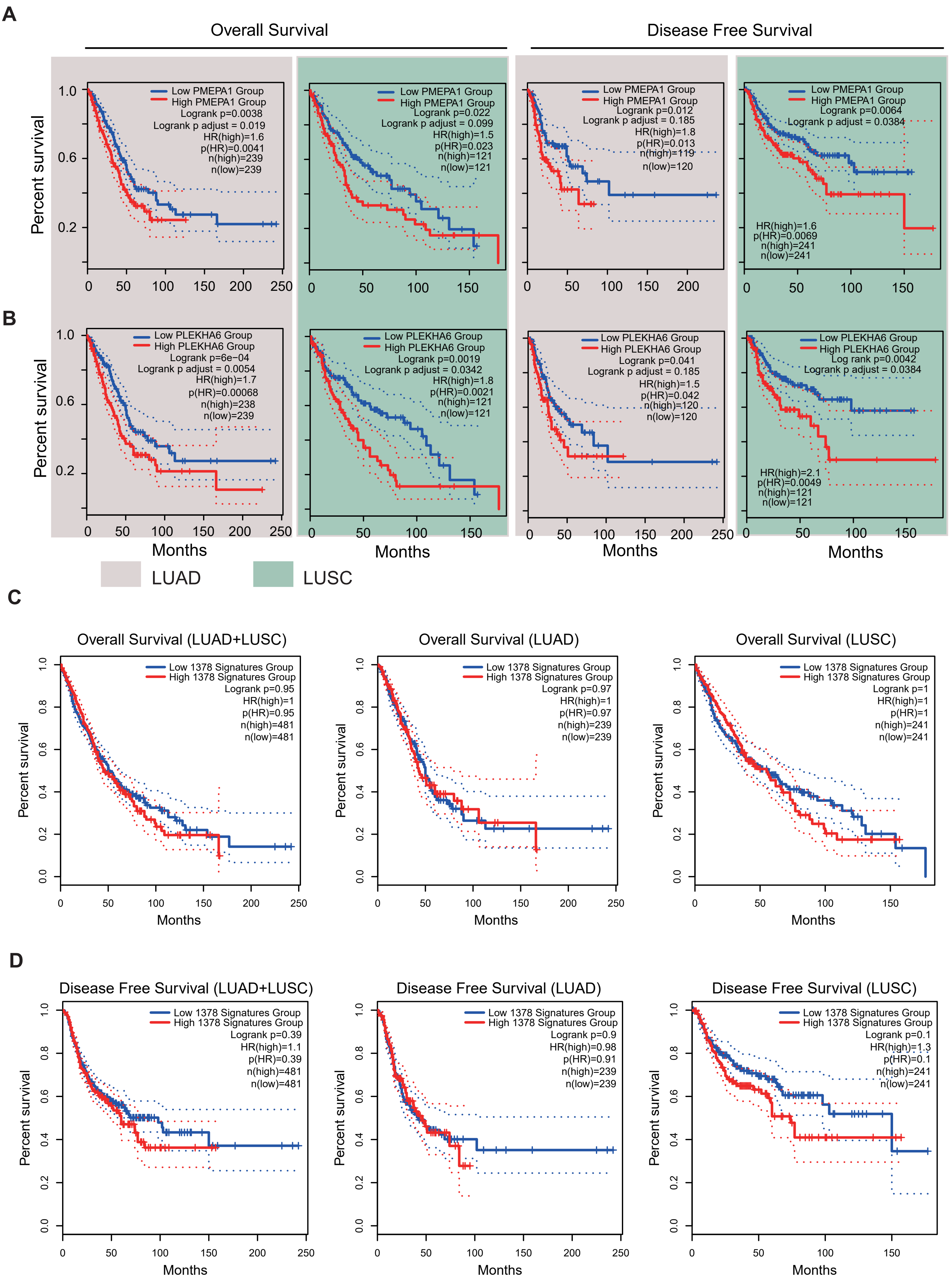
A
Overall Survival
Disease Free Survival
Low PMEPA1 Group
High PMEPA1 Group
Logrank p=0.0038
Logrank p adjust = 0.019
HR(high)=1.6
p(HR)=0.0041
n(high)=239
n(low)=239
Logrank p=0.022
Logrank p adjust = 0.099
HR(high)=1.5
p(HR)=0.023
n(high)=121
n(low)=121
Logrank p=0.012
Logrank p adjust = 0.185
HR(high)=1.8
p(HR)=0.013
n(high)=119
n(low)=120
Logrank p=0.0064
Logrank p adjust = 0.0384
HR(high)=1.6
p(HR)=0.0069
n(high)=241
n(low)=241
Percent survival
B
Low PLEKHA6 Group
High PLEKHA6 Group
Logrank p=6e−04
Logrank p adjust = 0.0054
HR(high)=1.7
p(HR)=0.00068
n(high)=238
n(low)=239
Logrank p=0.0019
Logrank p adjust = 0.0342
HR(high)=1.8
p(HR)=0.0021
n(high)=121
n(low)=121
Logrank p=0.041
Logrank p adjust = 0.185
HR(high)=1.5
p(HR)=0.042
n(high)=120
n(low)=120
Log rank p=0.0042
Logrank p adjust = 0.0384
HR(high)=2.1
p(HR)=0.0049
n(high)=121
n(low)=121
Months
LUAD
LUSC
C
Overall Survival (LUAD+LUSC)
Low 1378 Signatures Group
High 1378 Signatures Group
Logrank p=0.95
HR(high)=1
p(HR)=0.95
n(high)=481
n(low)=481
Overall Survival (LUAD)
Logrank p=0.97
HR(high)=1
p(HR)=0.97
n(high)=239
n(low)=239
Overall Survival (LUSC)
Logrank p=1
HR(high)=1
p(HR)=1
n(high)=241
n(low)=241
D
Disease Free Survival (LUAD+LUSC)
Logrank p=0.39
HR(high)=1.1
p(HR)=0.39
n(high)=481
n(low)=481
Disease Free Survival (LUAD)
Logrank p=0.9
HR(high)=0.98
p(HR)=0.91
n(high)=239
n(low)=239
Disease Free Survival (LUSC)
Logrank p=0.1
HR(high)=1.3
p(HR)=0.1
n(high)=241
n(low)=241
